# Independent representations of self-motion and object location in barrel cortex output

**DOI:** 10.1371/journal.pbio.3000882

**Published:** 2020-11-03

**Authors:** Jonathan Andrew Cheung, Phillip Maire, Jinho Kim, Kiana Lee, Garrett Flynn, Samuel Andrew Hires

**Affiliations:** 1 Department of Biological Sciences, Section of Neurobiology, University of Southern California, Los Angeles, California, United States of America; 2 Neuroscience Graduate Program, University of Southern California, Los Angeles, California, United States of America; Ecole Polytechnique Federale de Lausanne, SWITZERLAND

## Abstract

During active tactile exploration, the dynamic patterns of touch are transduced to electrical signals and transformed by the brain into a mental representation of the object under investigation. This transformation from sensation to perception is thought to be a major function of the mammalian cortex. In primary somatosensory cortex (S1) of mice, layer 5 (L5) pyramidal neurons are major outputs to downstream areas that influence perception, decision-making, and motor control. We investigated self-motion and touch representations in L5 of S1 with juxtacellular loose-seal patch recordings of optogenetically identified excitatory neurons. We found that during rhythmic whisker movement, 54 of 115 active neurons (47%) represented self-motion. This population was significantly more modulated by whisker angle than by phase. Upon active touch, a distinct pattern of activity was evoked across L5, which represented the whisker angle at the time of touch. Object location was decodable with submillimeter precision from the touch-evoked spike counts of a randomly sampled handful of these neurons. These representations of whisker angle during self-motion and touch were independent, both in the selection of which neurons were active and in the angle-tuning preference of coactive neurons. Thus, the output of S1 transiently shifts from a representation of self-motion to an independent representation of explored object location during active touch.

## Abstract 2

Skillful interactions with objects require knowledge of object location relative to self. This study of the mouse vibrissal system shows that during touch, primary somatosensory cortex output shifts from a representation of self-motion to the location of touched objects.

## Introduction

A major function of the mammalian cortex is to integrate sensory input with self-knowledge to form mental representations of the external world to guide flexible behavior [1,2]. Object location is one such representation, and it is essential for skillful navigation and object interaction [3–5]. Object locations can be rapidly and accurately identified via active touch [6–10]. In active touch, mechanosensory input is thought to be referenced to the movement and position of tactile sensors to produce a mental percept not of the self but of the object under investigation [2]. Determining where and how these sensory and motor signals are transformed by neural circuits into a representation of the external world would improve our understanding of brain function.

Head-fixed mice are an excellent model system to investigate the neural basis of object localization. They can locate objects along the anteroposterior axis of the face with submillimeter precision by sweeping a single whisker back and forth (i.e., whisking; [10]) and interpreting the mechanically evoked neural activity patterns transduced in the follicle that holds the whisker [11–13]. Similar sensorimotor mechanisms may underlie texture discrimination in rodents [14–16] and tactile sensing with tools in humans [17–18]. High-speed whisker imaging and mechanical models of whisker deformation provide rich knowledge of the sensory input and motor program underlying the computation of object location [19–24]. Early cortical processing of tactile input is topographically organized into columns of primary somatosensory cortex (S1) that have a one-to-one correspondence with large facial whiskers [25]. Intrinsic signal imaging allows whisker-specific neural activity to be targeted for electrical recording [26–27]. Furthermore, transgenic mouse lines allow assignment of observed neural activity patterns to neurons of specific types [28–29]. Thus, mice allow a dissection of self-motion and object-location representation at behavioral, perceptual, computational, and neural circuit levels.

A prime candidate for the construction of neural representations of object location are layer 5 (L5) pyramidal neurons of S1. S1 activity is required for whisker-based anteroposterior object localization [27] (though not object detection; [30]). L5 pyramids contain the major output of S1 to cortical and subcortical targets involved in decision-making, action selection, and motor control [29, 31–33]. Distinct cellular compartments of L5 pyramids receive sensorimotor features that are assembled in models of object-location representation and perception [10,34]. These features include sensory representations of self-motion from ventral posteromedial nucleus of the thalamus (VPM) [35–36] of touch from VPM and layer 3 (L3) and layer 4 (L4) of S1 [37–39] and efference copy from primary motor cortex (M1) [40–41]. Object location–specific calcium responses have been observed in tuft dendrites [42], apical trunk, and soma [43] of L5 pyramids. Thus, L5 pyramidal neurons have the appropriate inputs and outputs to transform sensation into an object-location representation that guides flexible behavior.

Here, we use single-unit juxtacellular electrophysiology to investigate the neural representation of sensory input, self-motion, and object location in L5 excitatory neurons during behavior. Over half of the active neurons encode self-motion during free-whisking, and a third encode the location of touched objects. This encoding does not require specialized training. Population responses to touch can decode object location with submillimeter accuracy. Contrary to expectations, the cellular identity and positional preferences of the touch-evoked object-location representation were uncorrelated with the self-motion representation during free-whisking. Thus, touch activates an independent representation of object location in L5, rather than amplifying an underlying representation of self-motion. These data suggest that a perceptual transformation from self to sensed object is accomplished by neural circuits interacting with L5 pyramidal neurons of S1.

## Results

### Experimental design

**Fig 1:**
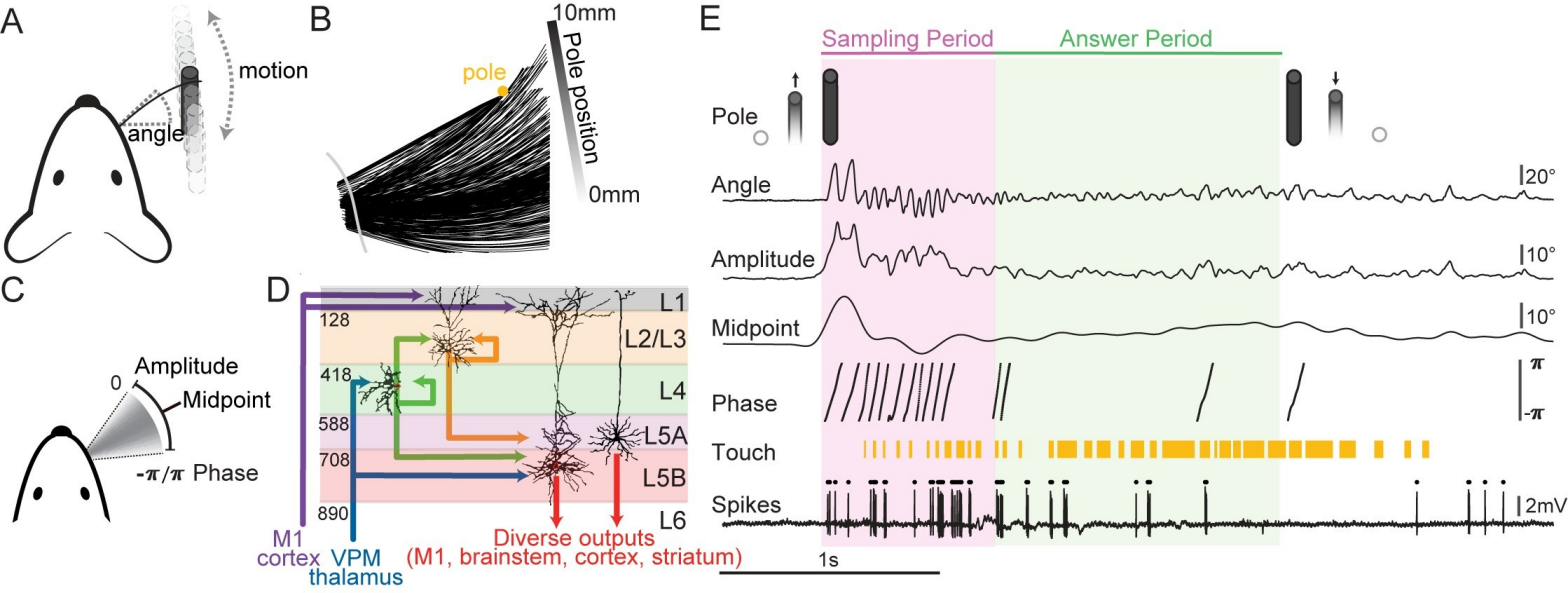
Head-fixed task and in vivo juxtacellular electrophysiology. (A) Schematic of task. Mice sweep a whisker forward and backward to locate a pole (black cylinder) presented along the anteroposterior axis. Angle is the azimuthal angle of the whisker at the follicle relative to the mediolateral axis of the animal. (B) Overhead view of whisker traces captured from a single trial. A mask (gray) crops traces near fur. (C) Angle time series can be decomposed to the Hilbert components amplitude, midpoint, and phase. (D) Selected excitatory flow into L5 neurons of S1 (*border depth in μm and dendritic arbors from Lefort and colleagues, 2009 [44]*). (E) Trial structure with example traces of recorded stimuli and spikes. Phase masked to periods of amplitude >5 degrees. Pole presentation is triggered 500 ms from trial start and takes approximately 200 ms to come into reach. Pole exits at varying times based on trial events. Data and code available at https://github.com/hireslab/Pub_S1LocationCode. L, layer; M1, primary motor cortex; S1, primary somatosensory cortex; VPM, ventral posteromedial nucleus of the thalamus.

To investigate the organization of neural representations in L5 during whisker-mediated exploration, we used variations of a go/no-go whisker-guided object-localization task in head-fixed mice [27]. Water-restricted mice (*n* = 16 VGAT-ChR2-EYFP mice) were trained to whisk and contact a smooth vertical pole presented randomly across a contiguous range (10 mm) of pole positions along the anteroposterior axis about 8 mm lateral from the whisker pad (Fig 1A). Mice were trimmed to a single whisker (C2) across all training and recording sessions. Whisker motion and object interactions were tracked from an overhead view at 1,000 frames per second (fps) (Fig 1B). Whisker traces were converted to time series of whisker azimuthal angle (i.e., angle), the Hilbert decomposition of amplitude, midpoint, and phase [41] and touch (Fig 1C). We serially recorded optogenetically tagged single neurons via blind juxtacellular loose-seal patch in and around layer 5B (L5B) of the C2 whisker representation of S1 (Fig 1D, S1A and S1B Fig; see Materials and methods). Each trial consisted of a 0.5-s pre-pole period, followed by a 0.75-s stimulus sampling period, and then a 1.25-s answer period in which licks triggered water dispensing or a brief time-out (Fig 1E). We recorded from 149 single units during active touch behavior. Twenty units were silent and 14 others were putative inhibitory neurons, based on short latency spiking in response to illumination of S1 with 473-nm light (S1C–S1E Fig), leaving 115 active putative excitatory neurons. Putative inhibitory units predominantly had shorter spike durations and more symmetric peak-to-trough heights (S1D–S1F Fig). To quantify the neural representation of sensorimotor features, we correlated these features to the times of detected action potentials.

### Representation of self-motion

**Fig 2:**
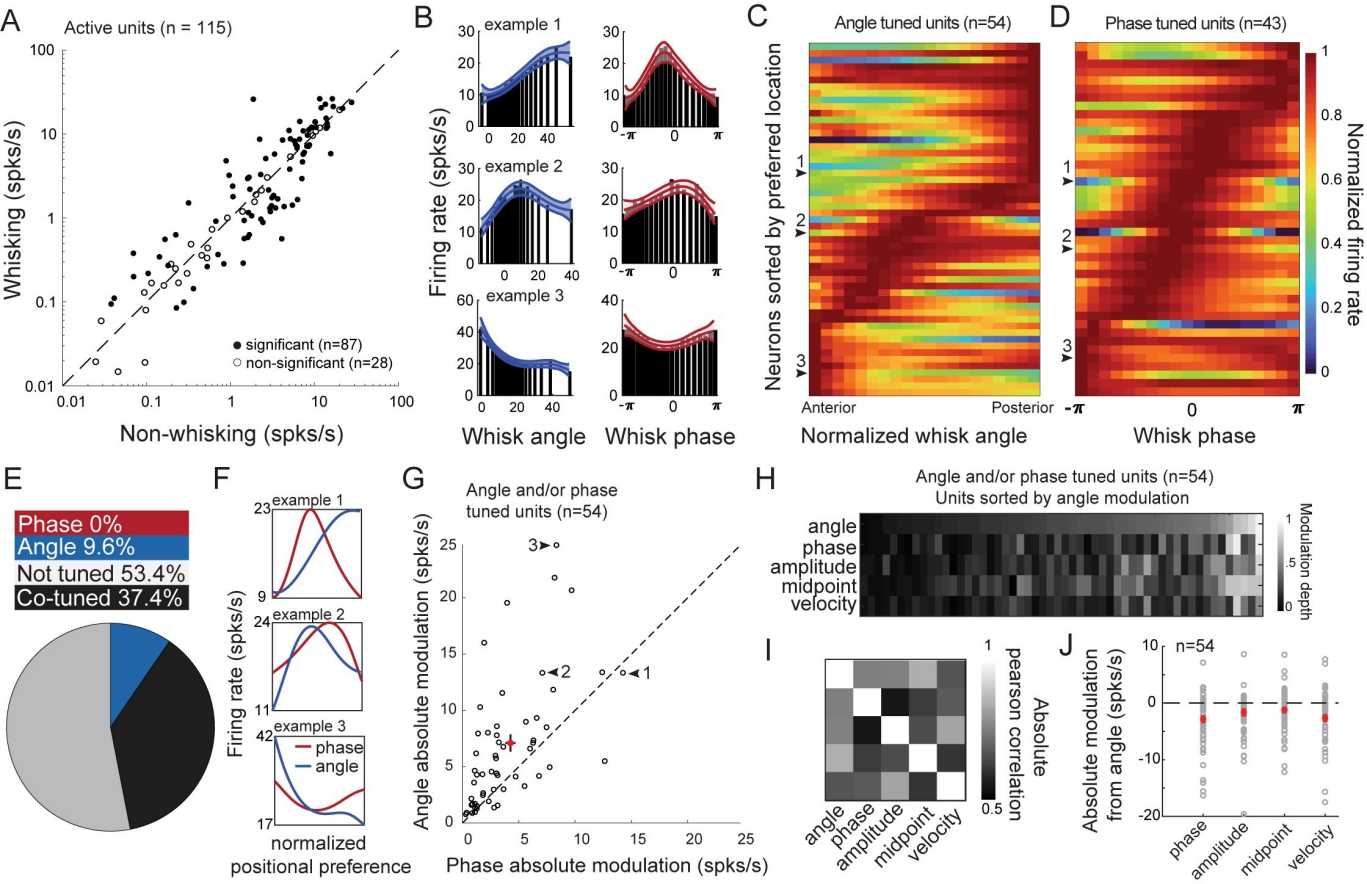
L5 excitatory neurons encode a representation of self-motion during free-whisking. (A) Firing rates for nonwhisking (4.7 ± 5.4 Hz) and whisking periods (5.3 ± 6.7 Hz) (*p* = 0.07, *t*-stat 1.8, df 114, paired-sample *t* test). Data are represented as mean ± SD. (B) Three example units tuned to both whisking angle (blue) and phase (red). (C) Population heat map for units tuned to whisker angle sorted by peak angle response. (D) Population heat map of phase-tuned units sorted by peak phase response. (E) Pie chart of self-motion tuning across the L5 excitatory population. Phase (red, 0/115), angle (blue, 11/115), co-tuned (black, 43/115), and not tuned to either (gray, 61/115). (F) Normalized positional preference for the three examples in (B): phase (red), angle (blue). (G) Absolute modulation depth (see Materials and methods) comparison between free-whisking phase and angle tuning. Red dot and error bars denote phase/angle mean ± SEM (4.0/6.8 ± 0.5/0.8 spks/s, *p* = 5.0e-5, *t*-stat = −4.42, df = 53; paired *t* test). (H) Modulation depth of angle, phase, amplitude, and midpoint for all angle-tuned units. (I) Contingency table of Pearson correlation coefficients for modulation depths across angle and motor variables. (J) Difference in absolute modulation between angle and motor variables (motor − angle modulation). Phase to angle (mean ± SEM = −2.8 ± 0.6, *p* = 8.8e-5, *t*-stat = 4.2, df = 53). Amplitude to angle (mean ± SEM = −1.6 ± 0.6, *p* = 2.2e-2, *t*-stat = 2.4, df = 53). Midpoint to angle (mean ± SEM = −1.1 ± 0.4, *p* = 1.4e-2, *t*-stat = 2.5, df = 53). Velocity to angle (mean ± SEM = −2.6 ± 0.6, *p* = 5.0e-5, *t*-stat = 4.4, df = 53). All compared using paired *t* test. Data and code available at https://github.com/hireslab/Pub_S1LocationCode. L, layer; spks/s, spikes per second.

We first examined the neural representation of self-motion during free-whisking (S2A Fig). Most neurons (87 of 115 active units) were significantly (chi-squared test) modulated (positively or negatively) by whisking, with the mean firing rate significantly increased from 4.7 ± 5.4 spikes per second (spks/s) (mean ± SD) during nonwhisking to 5.3 ± 6.7 spks/s (mean ± SD) during whisking (Fig 2A). The bulk of this increase occurred among neurons with nonwhisking firing rates in excess of 5 spks/s. Whisking-tuned neurons were relatively uniformly distributed across the recorded depth (S2B Fig). Since whisking was volitional [10], we could not dictate the exploration time or range of the mouse (S2C and S2D Fig), but many neurons (54/115) were significantly modulated with respect to whisker angle within the chosen range of whisking (Fig 2B and 2C). Across the population, preferred angles spanned the range of whisking (Figs 2C and S2E). Most of these neurons (43/54) were also modulated by phase in the whisker cycle (Figs 2B, 2D and S2F) with representations tiling the phase space. Across the population of 115 active putative excitatory units, 43 were tuned to whisker phase and angle, 11 to angle only, 0 to phase only, and 61 to neither (Fig 2E). Among neurons tuned to at least one, the mean depth of modulation to angle was significantly greater than to phase (*p* = 5.0e-5, Fig 2F and 2G, see Materials and methods). Greater modulation to angle was more correlated with greater modulation to whisking midpoint than to amplitude, phase, or velocity (Fig 2H and 2I). The absolute modulation depth of midpoint was most similar to that of angle (Fig 2J). This suggests that midpoint-correlated inputs are important for constructing an angle-tuned representation, which is consistent with the importance of midpoint in predicting choice during object localization [10]. These data show that during free-whisking, L5 excitatory neurons encode a representation of self-motion that is more specific to whisker angle than phase of whisk cycle.

### Representation of object location

**Fig 3:**
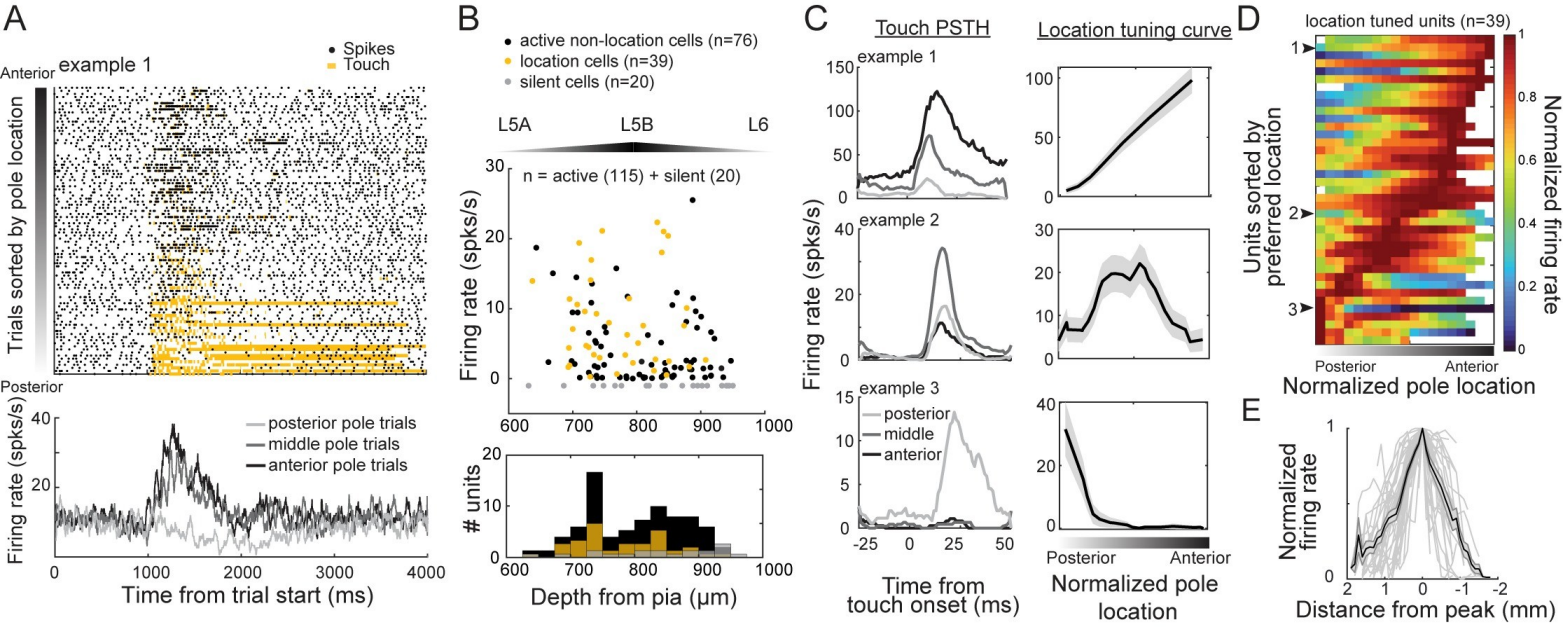
L5 S1 excitatory units are tuned to object location at touch. (A) Raster (top) and stratified PSTH by pole location (bottom) for example neuron tuned to anterior pole positions. (B) Average firing rate versus depth from pia for active non-location (black), location (gold), and silent (gray) putative excitatory units. (C) Touch PSTH (left) and location-tuning curves (right) for three example units tuned to anterior (top), middle (middle), and posterior (bottom) pole positions. Data are represented as mean ± SEM. (D) Population heat map of object location–tuned units, sorted by preferred location. White spaces are insufficiently sampled pole locations. (E) Shape of normalized tuning curves across all object location–tuned units. Data are represented as mean ± SEM. Mean half-max width response was 1.8 mm (approximately 9.2° of azimuth). Data and code available at https://github.com/hireslab/Pub_S1LocationCode. L, layer; max, maximum; PSTH, peristimulus time histogram; S1, primary somatosensory cortex; spks/s, spikes per second.

We then examined sensorimotor representations in the same neurons during active touch. Of the active putative excitatory neurons, 50 out of 115 were excited by touch. Touch responses were temporally sharp with short latency (Table 1, S3A Fig). In 39 of the 50 touch neurons, the number of spikes evoked was dependent on the anteroposterior position of the pole (Fig 3A). These touch location–tuned neurons were concentrated between 690 and 890 μm from pia (Fig 3B), roughly corresponding to L5B (Fig 1D, [44]). Touch-location tuning was driven by a greater probability of spiking and a greater number of spikes evoked per touch (S3B–S3D Fig). Touch location and maximum (max) curvature change were weakly correlated (mean Pearson’s r = 0.16 ± 0.13 SD, *n* = 115 sessions). To dissociate the potential effects of touch force from object-location tuning, we stratified angle-tuning curves into high and low max curvature halves in each angle bin. Stronger touches were associated with higher evoked firing rates than weaker touches (increase of 18.6% ± 2.2% SEM; *p* = 2.1e-4, *t*-stat 4.0, df = 49, one-sample *t* test), but object-location tuning was independent of touch strength (S3F–S3L Fig). Across the tuned population, the preferred object location spanned the entire range of touched pole locations (Fig 3C and 3D). The mean half-max width response was 1.8 mm (approximately 9.2° of azimuth) (Fig 3E). Thus, this subpopulation of L5 excitatory neurons form a distributed neural code for touched-object location.

**Table 1:** Table comparing properties of non-touch (*n* = 65), non-location-touch units (*n* = 11), and location-touch units (*n* = 39).

Quantified feature	Non-touch units (*n* = 65)				Non-location-touch units (*n* = 11)				Location-touch units (*n* = 39)			
	Mean	SD	Median	min–max	Mean	SD	Median	min–max	Mean	SD	Median	min–max
Whisking (Hz)	4.30	5.77	1.37	.01–23.86	4.09	4.54	2.39	.20–12.03	7.42	8.16	4.80	.06–26.33
Quiet (Hz)	4.15	5.21	2.01	.02–27.74	3.84	4.59	1.46	.07–13.84	5.87	5.84	3.26	.03–21.68
Proportion of spikes evoked by touch	0.19	0.17	0.14	.00–.72	0.35	0.23	0.27	.05–.72	0.42	0.24	0.40	.10–.99
Proportion of spikes evoked by touch and whisking	0.45	0.19	0.42	.10–.88	0.54	0.22	0.57	.18–.86	0.60	0.21	0.60	.16–.99
Touch onset latency (ms)					12.91	6.30	11.00	6.00–26.00	10.44	4.39	10.00	4.00–22.00
Touch-response duration (ms)					17.73	9.11	16.00	4.00–34.00	18.03	9.28	17.00	4.00–39.00
Spikes in response window (#)					0.41	0.34	0.40	.04–1.25	0.70	0.80	0.49	.06–3.60
Probability of touch response					0.30	0.20	0.30	.03–.64	0.40	0.28	0.39	.05–.93
Probability of response at peak bin					0.37	0.20	0.34	.07–.68	0.54	0.28	0.56	.14–1.00
Probability of response at trough bin					0.22	0.23	0.16	.00–.78	0.24	0.24	0.16	.00–.90
Response at peak bin (Hz)					25.12	10.81	21.58	11.48–40.82	51.17	36.51	41.12	6.55–154.97
Response at trough bin (Hz)					12.19	10.75	7.35	.00–30.16	18.33	21.47	11.86	.00–89.20

Abbreviations: max, maximum; min, minimum

**Fig 4:**
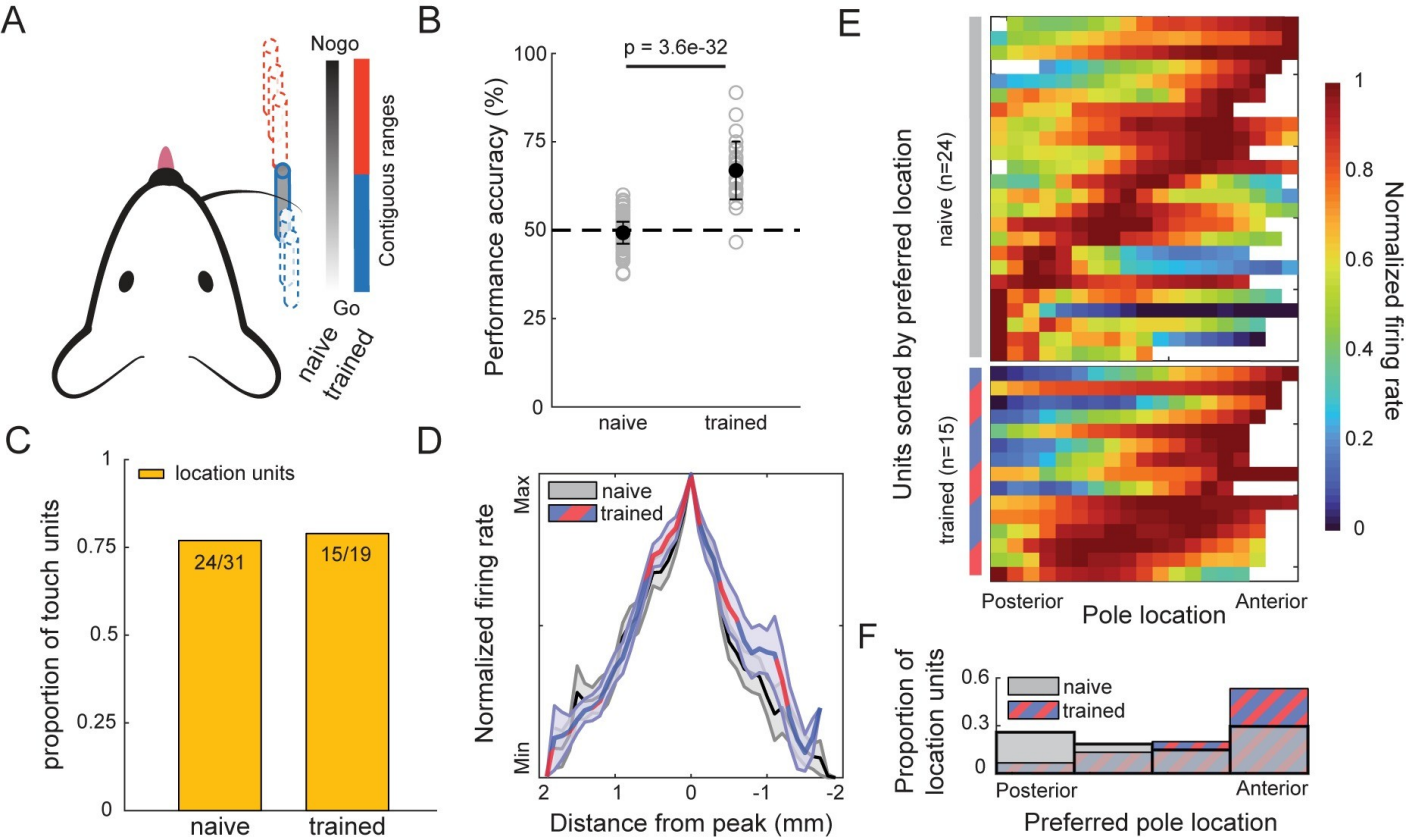
Object-location tuning does not require specialized training. (A) Schematic of two tasks. Mice were presented a pole randomly in a 10-mm range, 7–12 mm from the face. Naïve task, reward was available on 50% of trials, regardless of pole location. Trained task, reward exclusively available 100% of time in 0- to 5-mm proximal go range. (B) Performance on naïve (49.3% ± 3.2%, mean ± SD) and trained (66.9% ± 8.1%, mean ± SD) recording sessions (*p* = 3.6e-32, *t*-stat = 16.6, df = 113, unpaired *t* test). (C) Proportion of touch units that are location tuned for naïve (left; 77.4%) versus trained (right; 78.9%) animals. (D) Shape of normalized tuning curves for touch-location units from naïve (gray) and trained (red/blue) mice. (E) Population heat map of touch-location units from naïve (top 24 units) and trained (bottom 15 units) animals, sorted by preferred object location. Each row denotes a single location neuron. Bin resolution = 0.5 mm. (F) Histogram of positional preference of touch-location units compared between naïve and trained animals. (*p* = 0.10, *t*-stat = −1.7, df = 37; two-sample *t* test). Data and code available at https://github.com/hireslab/Pub_S1LocationCode. max, maximum; min, minimum.

Touch-location tuning did not require training in whisker-guided location discrimination. We performed recordings in two related tasks. In 85 naïve recording sessions with untrained mice (*n* = 10), water rewards were given randomly on 50% of the trials, regardless of pole location, whereas in 30 trained sessions, trained mice (*n* = 6) were first trained to discriminate go and no-go locations as in the study by Cheung and colleagues, 2019 [10] (Fig 4A and 4B), with water only available in the posterior go range. Trained mice made significantly more touches with less time spent whisking than naïve mice (S4A Fig). However, there was no significant difference in the proportion of touch-responsive units that were tuned to object location between naïve (*n* = 24/31, 77.4%) and trained (*n* = 15/19, 78.9%) (*p* = 0.27, Fisher’s exact test; Fig 4C), though we did observe a larger proportion of touch-responsive units in trained animals (S4B Fig). The width of the tuning was indistinguishable between the groups (Fig 4D), and the preferred locations spanned the full range of presented locations in both naïve and trained mice (Fig 4E and 4F).

**Fig 5:**
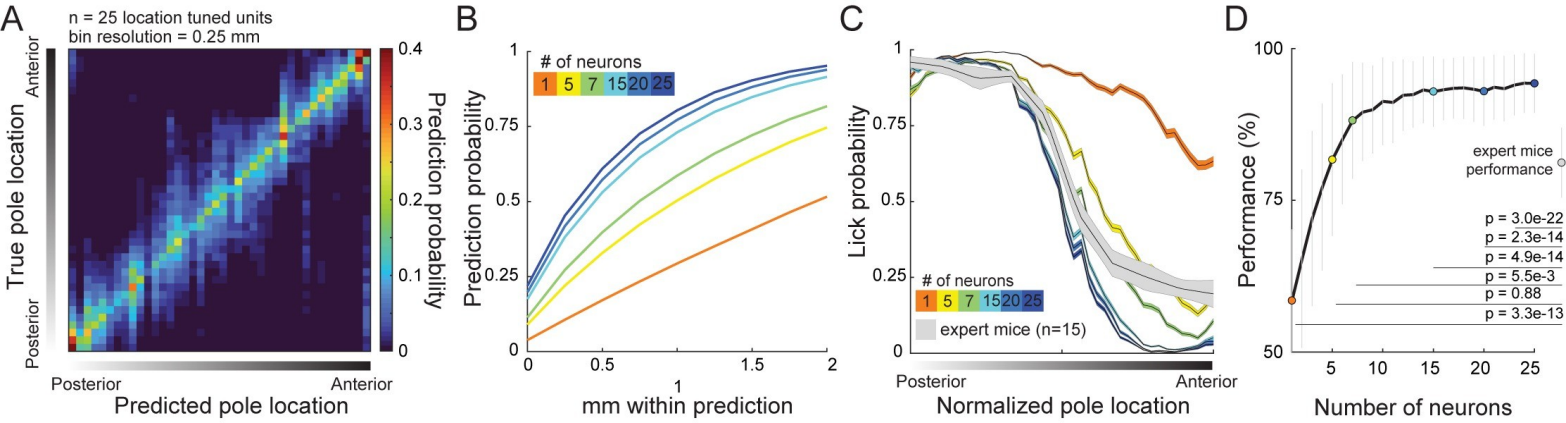
Object location is decodable to ≤0.5-mm precision from touch-evoked spike counts. (A) Contingency table of pole location decoding performance from 25 pooled unique touch-location units using a multinomial GLM. (B) Performance as a function of pool neuron count. (C) Average psychometric curves from 15 expert mice (gray; [10]) and neurometric curves from varying numbers of sampled location units. (D) Performance from neurometric curves compared to expert mice. Data are represented as mean ± SD. Solid black lines denote points significantly different (*p* < 0.05; two-sample *t* test) from expert-mouse performance. Data and code available at https://github.com/hireslab/Pub_S1LocationCode. GLM, generalized linear model.

To access location information from a distributed representation, downstream neurons must sample multiple members of the representing population. However, the number of possible inputs to a neuron is limited. Thus, we wondered how accurately the object location could be determined from varying numbers of randomly sampled object-location tuned neurons. We constructed a multinomial generalized linear model (GLM) to predict the location of the pole from the distribution of the number of spikes evoked by single touches (see Materials and methods, S3E Fig). A linear classifier pooling the touch-evoked spike counts from 25 of our location-tuned neurons (the subset with ≥75 touches in ≥80% of binned pole locations) predicted the pole location to ≤0.5-mm distance from actual on 60.5% ± 1.3% (mean ± SD) of touches (Fig 5A and 5B).

Our prior work showed that expert mice discriminate location to ≤0.5-mm resolution in this task [10]. How many location-tuned neurons are required to meet or exceed the psychometric performance of these expert mice? We constructed neurometric performance curves from the predicted object locations and compared them to mean psychometric curves from Cheung and colleagues, 2019 [10] (Fig 5C). Random sampling from five or more location-tuned neurons produced model performance that met or exceeded expert behavior (Fig 5D, see Materials and methods). This suggests that downstream neurons that sample from at least five location-tuned L5B neurons have access to a touch-by-touch object-location representation that meets or exceeds the behavioral performance of the mouse.

### Active touch evokes an object-location representation that is independent of self-motion tuning

**Fig 6:**
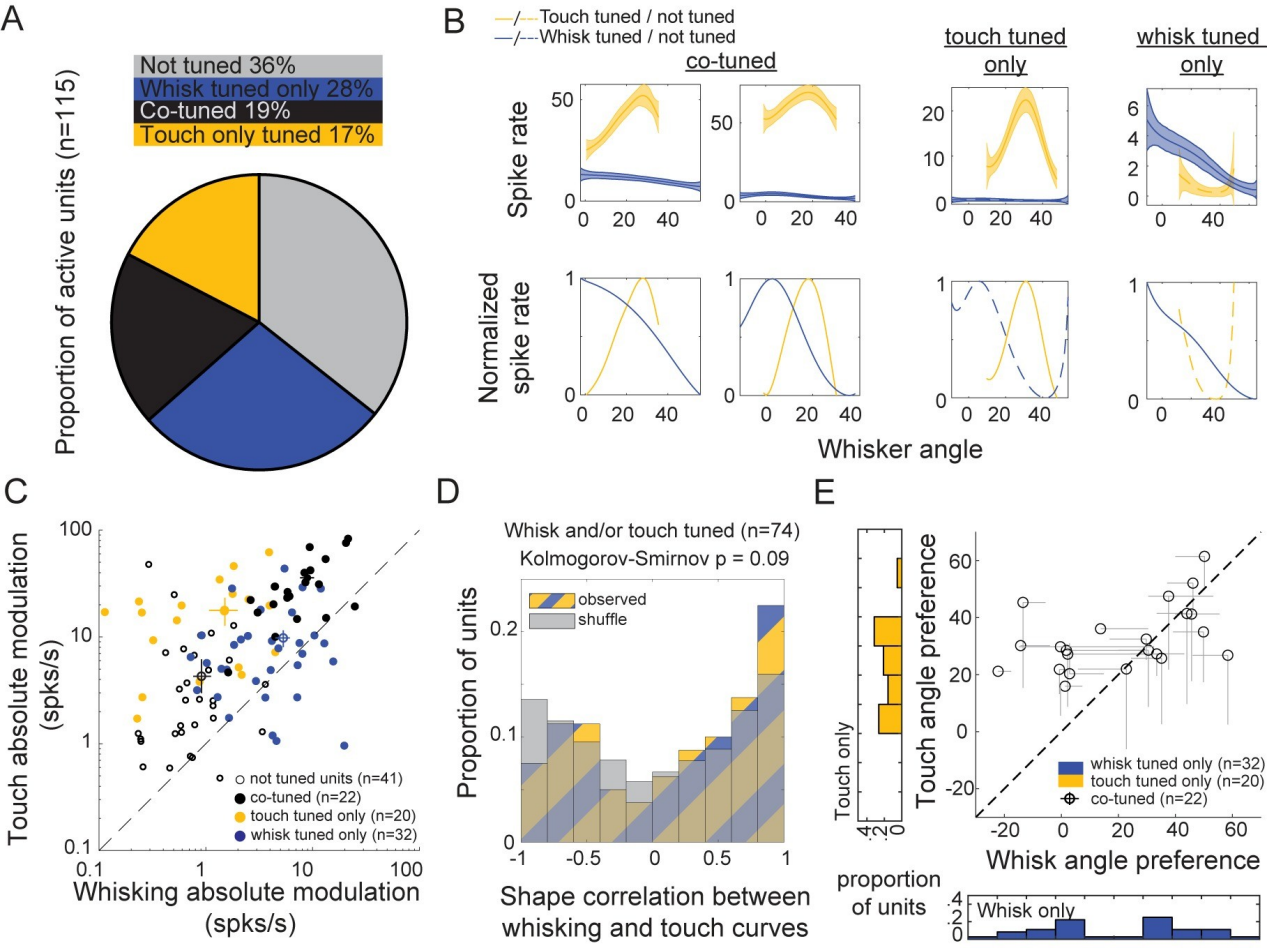
Active touch unmasks a distinct population code for object position in layer 5 of S1. (A) Proportion of units tuned to whisker angle during free-whisking (blue, 32/115), at touch (gold, 20/115), co-tuned (black, 22/115), or not tuned (gray, 41/115). (B) Absolute (top) and normalized (bottom) tuning curves for angle responses during free-whisking (blue) and at touch (gold). (C) Absolute modulation depth for angle tuning during free-whisking and touch for each class in (A). (D) Shape correlation between whisking- and touch-tuning curves for all units tuned to whisking and/or touch (blue and gold hash) compared to shuffled responses (gray). Kolmogorov-Smirnov *p* = 0.09. (E) Preferred angle during free-whisking versus at touch. Single-tuned units on histograms, co-tuned units on plot. Distance from midline for co-tuned units: (mean ± SD = 13.1° ± 11.1°, *p* = 1.7e-5, *t*-stat = 5.5, df = 21; one-sample *t* test). Data and code available at https://github.com/hireslab/Pub_S1LocationCode. S1, primary somatosensory cortex; spks/s, spikes per second.

Does touch amplify an underlying whisker-angle representation during free-whisking? Or does touch evoke an object-location representation that is independent of the free-whisking representation? Multiple lines of evidence support the independent model. First, 47% of neurons were tuned to angle during free-whisking, and 36% were tuned to angle during touch, but only 19% neurons were tuned to angle under both conditions (Fig 6A). Thus, tuning during free-whisking is neither necessary nor sufficient to exhibit angle tuning during touch. Moreover, this co-tuned overlap is nearly identical to an expected overlap (17%) if the two representations were independently distributed across the population.

We compared angle-tuned responses between free-whisking and touch in individual neurons using spike integration windows derived from each neuron’s touch-evoked response (Figs 6B and S5A). Whisker angle at touch is tightly correlated with (S5B Fig) and a proxy for the object location in this analysis (Fig 3, see Materials and methods). The average absolute modulation depth was 3.7× greater for touch (14.2 ± 1.7 Hz; mean ± SEM) than for whisking (3.8 ± 0.5 Hz; mean ± SEM) (Fig 6C). Note that since touch-evoked responses tended to be much larger, many neurons with higher absolute modulation to touch than whisking were not significantly touch-angle tuned (*p* < 0.01 analysis of variance [ANOVA] across angle bins). The shapes of normalized angle-tuning curves in each neuron were uncorrelated between the two conditions and not significantly different from a randomly shuffled population (Fig 6D). Finally, in co-tuned units, the angles of max response during free-whisking and at touch were significantly different (mean ± SD = 13.1° ± 11.1°, *p* = 1.7e-5, one-sample *t* test) and weakly correlated (Pearson’s r = 0.23) (Fig 6E). Repeating these analyses using phase instead of angle as the independent variable showed similar results (S6 Fig). We conclude that rather than amplifying an underlying tuning to whisker angle during free-whisking, active touch shifts population activity from a representation of self-motion to an independent representation of object location.

## Discussion

We quantified sensorimotor representations in L5 excitatory neurons during active whisker exploration and touch using juxtacellular electrophysiology (Fig 1). Most active neurons represented self-motion during free-whisking (Fig 2), with greater modulation by whisker angle than whisker phase. A third of L5 excitatory neurons were highly modulated by touched-object location (Fig 3). This location tuning did not require training (Fig 4). Pooling activity of five random location-tuned units discriminated object location with equal or better skill than expert mice (Fig 5), suggesting that neurons in downstream areas need only sample a handful of S1 outputs to access behaviorally relevant representations of object location. The representations of whisker angle and phase during free-whisking and at touch were uncorrelated at population and within-cell levels (Figs 6 and S6). Together, these data indicate that active touch shifts S1 output from a sensory representation of self-motion to a perceptual representation of object location.

### Limitations and advantages of the research

We note several limitations of our work. We primarily targeted recordings to L5B [44], where thick-tufted pyramidal neurons are more prevalent, using axial penetration distance from pia to estimate cell depth. However, cell types do not strictly respect layer boundaries, and this depth estimate is only accurate to within ±30 μm [45]. Because we recorded across multiple days during behavior, we did not attempt to recover cell morphology by juxtacellular filling. Thus, we could not differentiate cell types (e.g., thin- versus thick-tufted L5 pyramids, or L6A corticocortical cells [46]), their projection patterns (e.g., intertelencephalic [IT] versus pyramidal tract [PT]), or the extent to which whisker motion and object location tuning are segregated between these two classes as hypothesized in rat [47]. Half of object location–tuned units were also tuned to whisker angle during free-whisking, suggesting that these two features may not be cleanly divided between IT and PT cell types. Use of projection-specific L5 *cre*-lines (e.g., IT versus PT; [29]) could determine the extent to which self-motion and object-location representations are segregated by cell type and projection class in future work. Finally, we did not establish a causal role for location coding neurons in driving perceptual choice during object localization. Recent developments in structured illumination and optogenetics [48–49] may allow testing this in the future.

However, our approach also had several advantages over prior investigations of S1 activity during active whisker touch. Optogenetic tagging allowed us to identify putative excitatory versus inhibitory units. Juxtacellular loose-seal recording, considered a gold standard for extracellular single-unit isolation [50], allowed us to sample activity with high accuracy and temporal fidelity without bias from firing rate, avoid potential misassignment of synchronous touch-evoked spikes [39], and avoid false negative responses common in calcium imaging when scanning population-sized fields of view [51]. The high temporal resolution of electrophysiology allowed us to determine whisker angle and phase tuning during free-whisking (Fig 2) and its relationship to tuning at touch (Figs 3 and 6), which was not investigated in prior studies using calcium imaging [42–43].

### The transformation from self-motion to object-location representation

Contrary to our expectations, we found that within L5, the cellular identity of angle-tuned neurons (Fig 6A and 6C) and their angle preferences (Fig 6B and 6E) were uncorrelated between free-whisking and at touch. The same was true for phase tuning (S6 Fig). This was surprising, because prior stereotrode recordings across layers of rat S1 showed touch responses that were highly amplified when they occurred at the peak of free-whisking phase tuning [52]. Moreover, the preferred phase during free-whisking and at touch were tightly correlated. The reason for our differing results is unclear. Our mice were head-fixed, whereas rats could crane their head, which could introduce head-direction effects on neural coding. There could be species-specific differences in phase, position, or touch encoding. Potentially supporting this notion, a recent report found no relationship between phase preference of stick-slip response and of surface or air whisking in mouse S1 [53]. A more tantalizing possibility is that the encoding of touch responses with respect to whisking features changes across the layers of S1. We targeted recordings to excitatory neurons of infragranular output layers, whereas Curtis and Kleinfeld's touch neurons were mostly found in granular input and deep infragranular layers. Phase tuning from reafferent input is prevalent in granular L4 [36, 39, 54], whereas the morphology [55] and circuit connectivity of infragranular L5 pyramids [38] give them access to internally generated whisking envelope signals [40]. Neural tuning for the azimuthal angle of touched objects has been hypothesized to arise from combining whisking phase and envelope signals with touch at an unspecified location [34]. Our data suggest that object-location tuning emerges from integration of touch and whisking signals within or prior to L5 in S1.

How is the transition from self-motion to object-location representation accomplished? At least three mechanisms could play a role. First, touch-induced follicle stresses differ from those during free-whisking [12], so distinct patterns of mechanosensory transduction [13] likely underlie at least part of output shift. Second, touch and whisking are encoded by largely distinct populations in superficial layers [56], which project to L5B [44, 57]. Thus, touch recruits a new set of interlaminar S1 projections that influence L5 responses. Third, touch could enhance integration of distant inputs on L5 dendrites [43] by transient changes in dendritic conductances [58]. M1 input is strongest in electrically distant tuft dendrites of L5 neurons [38]. Thus, touch could transiently increase the influence of efference copy from M1 on L5 activity, further contributing to the distinct representation. Determining the extent to which each of these possible mechanisms contribute to object-location tuning in L5 of S1 may reveal more general principles for how the transformation from sensation to perception is accomplished by cortical circuits.

## Materials and methods

### Lead contact and materials availability

Further information and requests for resources and reagents should be directed to and will be fulfilled by the lead contact, Samuel Andrew Hires (shires@usc.edu).

### Ethics statement

All procedures were approved under USC IACUC protocols 20169 and 20788 in accordance with United States national guidelines issued by Office of Laboratory Animal Welfare of the National Institute of Health.

### Experimental model and subject details

Sixteen VGAT-ChR2-EYFP mice (JAX B6.Cg-Tg), both male and female, of at least 3 mo of age were used for the following experiments. A complete description of the head-plate procedure has been documented in previous work [59]. Postoperatively, mice were housed with littermates or singly housed if fighting occurred. Mice were provided food ad libitum and water restricted to 1 mL per day for 1 wk before training and recording. A daily health and weight assessment was completed to ensure mice were healthy.

### Method details

#### Object-localization task

Mice were trained in a whisker-based go/no-go object-localization task. Using a single whisker (C2), water-restricted mice were motivated to whisk and identify the location of a smooth vertical pole (0.6-mm diameter) 7–12 mm lateral from the whisker pad. The pole moved along the anteroposterior axis across 10 mm and was positioned using stepper linear actuators with 99-nm resolution, 25-μm accuracy, and <5-μm repeatability (Zaber NA11B30-T4). To avoid potential ultrasonic cues associated with stepper motor movement, the pole was jittered 0–127 microsteps (0–25 μm) on each trial. A pneumatic linear slider (Festo) was used to raise the pole vertically into touch reach for each trial. The Festo also provided a sound cue on pole presentation onset.

Specific pole locations rewarded mice with water (4–8 μL), punished mice with a time-out (2 s), or had no effect based on the mouse’s decision to lick or withhold licking. In a go/no-go paradigm, four trial outcomes exist. In a minority of sessions in which the animals were trained, the close posterior 5 mm of pole locations (go) were rewarded with water rewards upon licking (hit) or had no effect if mice withheld licking (miss). The far anterior 5 mm of pole locations (no-go) were punished with time-out (false alarm) or had no effect if mice withheld licking (correct rejection). For the remaining sessions, rewards and punishment were given regardless of the pole location—go trials and no-go trials had overlapping pole locations.

#### Behavior, videography, and electrophysiology

Animal behavior, videography, and electrophysiology were synchronized and captured during task performance using EPHUS (https://www.janelia.org/open-science/ephus). A single computer running BControl (MATLAB 2007b) was used to initiate each trial of the object-localization task and synchronize video and electrophysiology recordings via a second computer running EPHUS. Trial onset triggered high-speed video capture of whisker motion (1,000 fps) and electrophysiology recording of single-unit activity (MultiClamp 700b).

Whisker motion was captured from an overhead view and spanned 4 s, spanning the period prior to pole onset to response window. Video frames were acquired using Basler acA200-340kmNIR camera and Edmund Optics 0.18X 1⁄2” GoldTL Telecentric Lens (Model # 52–258) under 940-nm illumination on Streampix 6 software. Whisker shape and position were traced and tracked using Janelia Farm’s Whisker Tracker (https://www.janelia.org/open-science/whisk-whisker-tracking). A mask was traced around the edge of the fur to reduce tracking noise. Whisker angle is quantified at the intersection between the mask and the whisker. The whisker midpoint, instantaneous phase, and amplitude were decomposed from the bandpass- and zero phase–filtered (6–60 Hz, Butterworth) whisker-angle time series using the Hilbert Transform (MATLAB 2018b: hilbert). Whisking amplitude and phase are defined as the magnitude and phase angle (radians) of the Hilbert Transform of the whisker-angle time series, respectively. A phase value of 0 is the most protracted location of the whisk cycle, π and −π are the most retracted position in that cycle, and the sign of +/− define retraction or protraction whisking directions. Whisking midpoint is the filtered (6–60 Hz) difference between whisker-angle time series and bandpass-filtered signal. Whisker curvature is the amount of bending of the whisker measured 3–5 mm lateral from the whisker mask.

The precise millisecond of touch was determined through custom MATLAB software using distance to pole and change in whisker curvature. This was followed with manual curation of images of uncertain whisker and pole intersections.

#### In vivo loose-seal juxtacellular recordings

All animals used in this study were adult male or female transgenic mice (VGAT-ChR2-EYFP) expressing channelrhodopsin in inhibitory units. Following head-plate surgery, mice were trimmed to one whisker (C2), and intrinsic signal imaging was used to target the barrel column associated. A single whisker was maintained throughout training and recording. Prior to recording, animals were anesthetized (2% isofluorane) and a small craniotomy (200–300 μm) was made above the barrel column associated with the C2 whisker. On the first day of recording, animals were allowed to recover for 1 h before recording. Recordings were repeated for 4.8 ± 1.5 sessions (mean ± SD) per animal.

To sample single-unit spiking activity in a manner unbiased by firing rate, blind juxtacellular loose-seal patch recordings were targeted to L5 (600–950 μm from pia [44]) neurons using patch pipettes (Warner Instruments; 5–8 MΩ) filled with 0.9% saline (Growcells). Electrical recordings (*n* = 149 neurons) were acquired and amplified using MultiClamp 700b and Headstage CV-7B. The pipette axis was aligned parallel to the C2 barrel column at 35°. To perform an unbiased sampling of L5, we recorded from any isolated unit. An isolated unit was identified by an increase in resistance to 15–20 MΩ. Once a unit was isolated, 10 trials of the behavioral task was run to test for spikes during performance. If spikes were observed, an isolated unit was maintained for at least 100 trials (137 ± 57; mean ± SD). Upon recording completion, 10 trials of a 10-Hz pulse of blue light (473 nm, 10 pulses for 20 ms each at 15–20 milliwatts, beam width 200-μm diameter at skull, UltraLasers Model CST-L-473 nm– 50—OEM) focused onto the recording site from overhead was used to test whether the recorded unit was an interneuron. Short latency spiking (or inhibition) to the light pulse indicated if the neuron was putative inhibitory (or excitatory) (S1C Fig). Fourteen units were putative inhibitory and excluded from analysis. The spike waveforms of these units clustered (S1F Fig) but were not cleanly separable from the excitatory population based on waveform alone. This is consistent with the diversity of inhibitory cell types in barrel cortex and with possible cell-type misidentification due to inhibitory network effects. On the other hand, if an isolated unit did not spike after 10 trials, a current pulse (100 μs, 20 nanoamps) was injected to check if a unit was attached. If a burst of spikes was observed, we deemed that neuron a silent cell.

#### Histology

DiI (ThermoFisher D282) was coated onto a patch pipette and inserted into the recording location on the final day of recording to identify the location of recordings. DiI-coated pipettes were inserted 1,000 μm deep into the recording location and left there for 5 min to ensure proper coating of the recording location. Two hours post dye, animals were deeply anesthetized with ketamine (110 mg/kg) and xylazine (10 mg/kg) cocktail before perfusion with 0.1 M sodium phosphate buffer, followed by 4% paraformaldehyde (PFA, in 0.1 M sodium phosphate buffer). The fixed brain was then flattened along the axis perpendicular to the barrel column.

The flattened brain was immersed in 4% PFA for 1 h post perfusion and transferred to 20% sucrose solution for 1 d and then 30% sucrose for 1 d. Slices (100 μm) were cut tangentially and cytochrome oxidase staining was performed to reveal the barrel columns. Fluorescence imaging was done to recover the location of the DiI track. Recording location was determined by overlapping fluorescent track on top of bright-field imaging of barrel columns.

### Quantification and statistical analysis

#### Defining touch-response window

A smoothed (Bayesian adaptive regression splines [BARS]; [60]) response −50 ms to 50 ms around touch was used to evaluate the touch-response window. The touch-response window is defined as any time point from 5 to 50 ms post touch in the smoothed response that exceeded baseline (−50 to 0 ms pre-touch) ± the 95% confidence interval. Two criteria were imposed to ensure an accurate response window was captured: (1) the mean firing rate of the touch response had to be >2 Hz; (2) the touch-response window had to be >4 ms. A touch neuron is defined as any neuron that had a touch-response window.

#### Tuning curves

A tuning curve is the response (firing rate) as a function of a stimulus (e.g., whisker position). For a single neuron, 5% of sampled touches or 5% of total whisking time points were used to define a point along the touch- or whisking-tuning curve. This method ensured 20 equally sampled bins consisting of stimulus (e.g., whisker position) and response (firing rates) values. For touch tuning, the response is defined as the firing rate within the touch-response window as defined above. For whisking tuning, the same response window as touch was used. If a neuron was not tuned to touch, the median touch-response window across all neurons was used to evaluate whisking tuning. The median touch-response window is 10–28 ms post touch. The stimulus value is defined as the median of the stimulus in each sampled bin. Response values are defined as the mean of the responses in each sampled bin. Tuning curves were generated by smoothing using BARS on the stimulus and response values. Neurons that had mean whisking responses less than 2 Hz were not evaluated.

To define whether a neuron was tuned to a specific location, we used a two-step process. We first performed a one-way ANOVA at alpha level of 0.01 to identify if any position’s firing rate at touch or during free-whisking was significantly different from another. If a neuron passed this first test, we moved onto the second step of the evaluation. In the second step, we shuffled touch/whisking responses 1,000 times and evaluated F-values from a one-way ANOVA. If the observed F-value was above the 95th percentile of the shuffled population distribution of F-values, we deemed the neuron as tuned. This second evaluation further ensures that the tuning we observed was not due to noise in neural responses. A neuron was considered location-tuned if it passed both tests.

Tuning preference is the location of the peak response of the tuning curve. To define the width of the tuning, a multiple comparison test using a Tukey-Kramer–type critical value was used to identify the first bins in both directions that were significantly different from the peak value. If no bins were significant, no modulation width was defined. Max and min responses were calculated from BARS-fitted tuning curves.

In computing tuning curves using whisker angle at touch instead of object location, we find that two more units qualify as tuned (S5B and S5C Fig). The two units exhibit a nonlinear second-order polynomial relationship between whisker angle and pole location. This second-order polynomial fit leads to nonlinear increases in whisker angles for incremental gains in pole location, causing those two units to have tuning to far locations not seen when observing pole locations.

#### Modulation

The absolute modulation depth and modulation depth for each tuning curve are calculated as:

absolutemodulationdepth=maxresponse−minresponse

$$modulation\;depth=\frac{\max\;response-\min\;response}{\max\;response+\min\;response}$$

#### Neural decoding

We used multinomial logistic regression to decode pole location implemented using glmnet [61]. Only touch units that sampled at least 80% of the pole location range were used for decoding. Each unit had a tuning curve that was interpolated to 40 bins to estimate location to 0.25-mm resolution. At each bin, 50 samples were drawn from a Poisson pdf with a λ as the mean of each interpolated bin. We justified drawing from a Poisson pdf because we found that at touch the number of spikes generated in the touch-response window followed a Fano factor of 0.94 ± 0.22 (mean ± SD, S3E Fig). For the design matrix, each row is a location bin, each column a single neuron, and each entry a sampled neural response for the associated neuron.

The decoder was run for 10 iterations. During each iteration, a random 70% of trials were allocated for training and the remaining 30% for test. Lasso regularization (alpha parameter 0.95) was used to reduce overfitting. To identify the number of units required, we sampled varying numbers of neurons with replacement from the units used to train the original model 500 times. The indices of the selected neurons were used to create a new population design matrix and matrix of learned coefficients from the original design matrix and learned coefficients. The prediction probabilities of location were computed by the below:

$$h_{\theta }(x)=g(\theta^{T}x)$$

$$\mathrm{where}\;g(z)=\frac{1}{1+e^{-z}}$$

where *h_θ_*(*x*) is the hypothesis function, *θ^T^* are the learned coefficients, *x* is the input design matrix, and *g*(*z*) is the normal function of logistic regression used to calculate prediction probabilities.

The predicted location was chosen as the location with the highest probability. Model evaluation of accuracy and resolution was performed on the test set. Model accuracy is defined as the total number of correct predictions divided by the total number of predictions. A confusion matrix made from true and predicted locations was normalized across the total number of given true cases and used to define the decoding resolution and neurometric curves. Decoding resolution is defined as the total number of predictions within *n* bins of the diagonal, where each bin was 0.25 mm. Neurometric curves, defined here as the choice to lick given neural activity, is defined as the sum of predictions along true values for the go predictions (left half of the confusion matrix). Simulated neurometric curve performance for licks were defined as any lick probability that exceed 50%.

## Supporting information

S1 Fig Recording targeting, recovery, and opto-tagging. (A) Intrinsic signal imaging highlighting region of activity during whisker stimulation (top) overlaid with skull vasculature. (B) A 4× (top) and 10× (bottom) zoom of recovered DiI on top of cytochrome oxidase labeling of barrel field. (C) Example trace of single stimulation (480 nm, 10-Hz pulse) trial (top) with zoom of first 500 ms of stimulation (bottom). (D) Average spike waveforms of five putative excitatory and inhibitory neurons. (E) Group statistics of putative excitatory and inhibitory waveforms. (F) Individual waveform statistics for putative excitatory and inhibitory neurons.
(TIF)

S2 Fig Whisker angle during free-whisking tuning. (A) Example whisker trace with spikes overlaid for one example cell tuned to whisker angle during free-whisking. (B) Average firing rate and depth from pia for active non-location (black), whisking angle (blue), and silent (gray) units. (C) Scatter of mean ± SD (1.4 ± 0.5) for time (seconds) spent whisking for each recorded neuron. (D) Cumulative distribution function of whisker-angle sampling during free-whisking for all recorded units (gray) and population average ± SEM (red). (E) Free-whisking angle tuning across the population of significantly tuned units (*n* = 54). (F) Phase preference with modulation depth (Materials and methods) across the population of phase-tuned units (*n* = 43).
(TIF)

S3 Fig Whisker angle during touch tuning. (A) Three example touch units and their responses from touch onset. (B) Cumulative distribution function showing the number of touches made for each recording session (gray, *n* = 115) and the population average ± SD (red). (C) Response probability of generating a response above baseline ± 95% CI in the most preferred location versus the least preferred location (*p* = 1.9e-14, *t*-stat = 11.9, df = 38, paired *t* test). (D) Same as (C) but for the firing rate of responses (*p* = 7.6e-11, *t*-stat = 8.9, df = 38, paired *t* test). (E) Justification for modeling spikes using a Poisson process. Black dots denote scatter of spike count (0.73 ± 0.81, mean ± SEM) against Fano factor (0.94 ± 0.24, mean ± SEM) for each point along the angle at touch-tuning curve (*n* = 784 points). Red dots denote average for each individual location at touch-tuned neuron. (F) Population (*n* = 39 units) difference between high and low curvature touches controlling for position (*p* = 0.98, one-way ANOVA). (G) Example unit protraction touches (*n* = 404) highlighting relationship between whisker angle at touch and max curvature of the touch. (H) Single example unit comparing angle-tuning curves for the top 50% of curvature changes (i.e., high) versus the bottom 50% of curvature changes (i.e., low). (I) Heat maps of positional tuning for the same example neuron in (F) and (G) during high (left) and low (right) curvature changes. (J-L) (G), (H), and (I) for another example unit. ANOVA, analysis of variance; max, maximum.
(TIF)

S4 Fig Naïve versus trained animals comparison. (A) Comparison of number of touches made per trial (left, *p* = 2.5e-3) and proportion of time whisking (right, *p* = 2.6e-6) between naïve (gray) and trained animals (red/blue hash). Both compared using two-sample Kolmogorov-Smirnov test. (B) The distribution of non-touch units, touch-location units, and touch non-location units compared between recordings from naïve (*n* = 85) and trained (*n* = 30) animals
(TIF)

S5 Fig Co-tuning of whisker angle during free-whisking and touch. (A) Tuning curves with observed firing rates (top) and normalized firing rates (bottom) for co-tuned (*n* = 22), touch-tuned only (*n* = 20), and whisking-tuned only units (*n* = 32). Solid lines and dashed lines denote tuning and not tuning, respectively. (B) Whisker angle at touch is tightly correlated with anteroposterior object location. Three example sessions are shown. (C) Population heat map of angle-tuned units, sorted by preferred angle at touch. White spaces are insufficiently sampled pole locations.
(TIF)

S6 Fig Co-tuning of whisker phase during free-whisking and touch. (A) Pie chart highlighting proportion of units phase tuned at whisking (maroon, 20/115), at touch (gold, 25/115), co-tuned (black, 23/115), or not tuned (gray, 47/115). (B) Tuning curves with observed firing rates (top) and normalized firing rates (bottom) for co-tuned (*n* = 23), touch-tuned only (*n* = 25), and whisking-tuned only units (*n* = 20). Solid lines and dashed lines denote tuning and not tuning, respectively. (C) Absolute modulation depth for angle tuning during free-whisking and touch for each class in (A). Average absolute modulation depth was 7× greater for touch (16.5 ± 1.9 Hz; mean ± SEM) than for whisking (2.3 ± 0.3 Hz; mean ± SEM). (D) Shape correlation between whisking- and touch-tuning curves for all units tuned to whisking and/or touch (maroon and gold hash) compared to shuffled responses (gray). Kolmogorov-Smirnov *p* = 0.11. (E) Scatter of preference during free-whisking and touch for co-tuned units (mean ± SD; 0.7 ± 0.5 radians, *p* = 1.7e-6, *t*-stat = 6.4, df = 22; one-sample *t* test). Histograms denote phase preference for units tuned to either touch or free-whisking phase.
(TIF)

## Abbreviations

ANOVA: analysis of variance
fps: frames per second
GLM: generalized linear model
IT: intertelencephalic
L3: layer 3
L4: layer 4
L5: layer 5
L5B: layer 5B
M1: primary motor cortex
max: maximum
PSTH: peristimulus time histogram
PT: pyramidal tract
S1: primary somatosensory cortex
spks/s: spikes per second
VPM: ventral posteromedial nucleus of the thalamus
