# Supplementary figures and images for: Independent representations of self-motion and object location in barrel cortex output
Source: PLoS Biol. 2020 Nov 3;18(11):e3000882. doi: 10.1371/journal.pbio.3000882 (PMC7665803; doi:10.1371/journal.pbio.3000882)

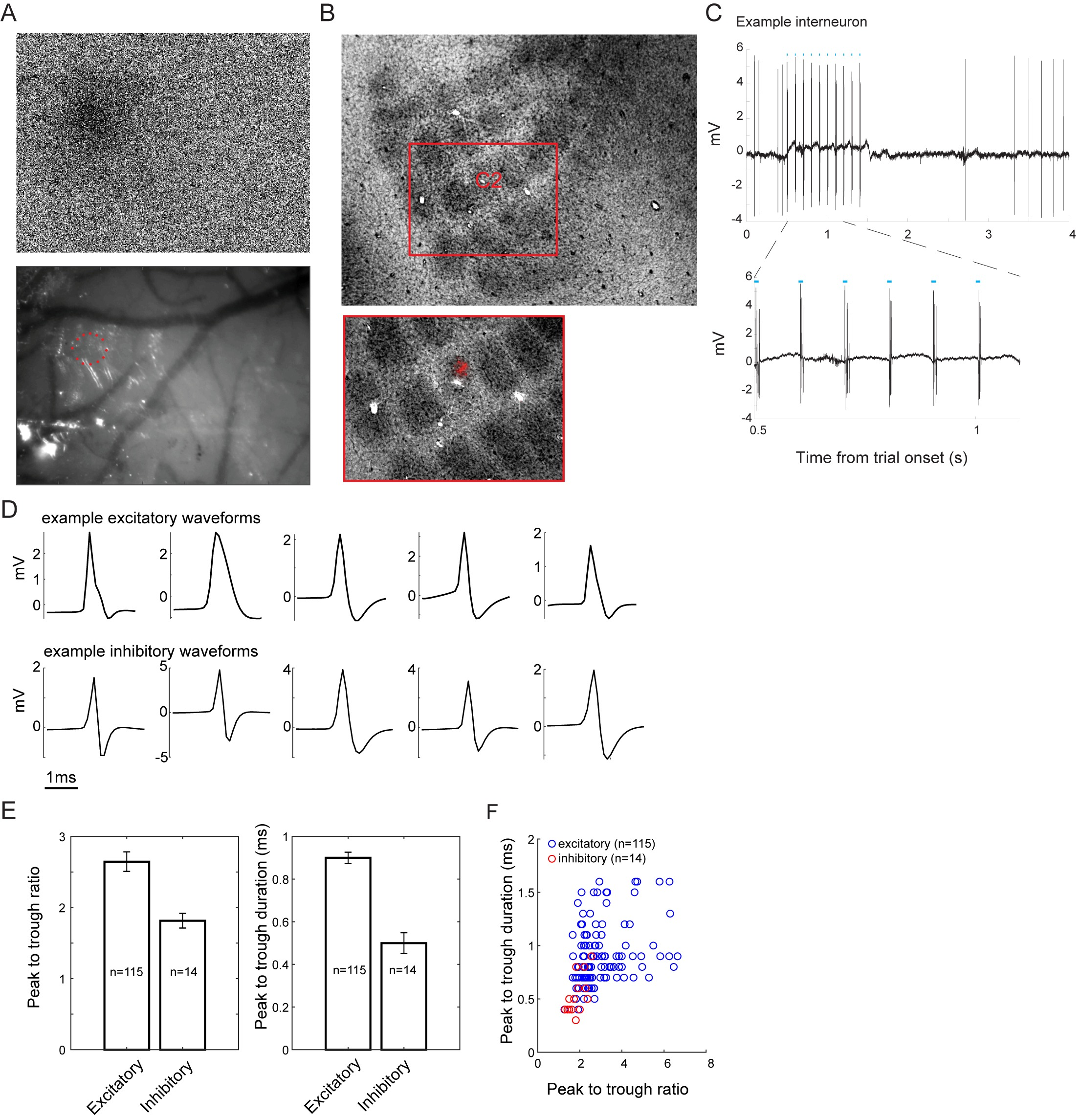

Supplement: S1 Fig — (A) Intrinsic signal imaging highlighting region of activity during whisker stimulation (top) overlaid with skull vasculature. (B) A 4× (top) and 10× (bottom) zoom of recovered DiI on top of cytochrome oxidase labeling of barrel field. (C) Example trace of single stimulation (480 nm, 10-Hz pulse) trial (top) with zoom of first 500 ms of stimulation (bottom). (D) Average spike waveforms of five putative excitatory and inhibitory neurons. (E) Group statistics of putative excitatory and inhibitory waveforms. (F) Individual waveform statistics for putative excitatory and inhibitory neurons. (TIF) [file pbio.3000882.s001.tif]

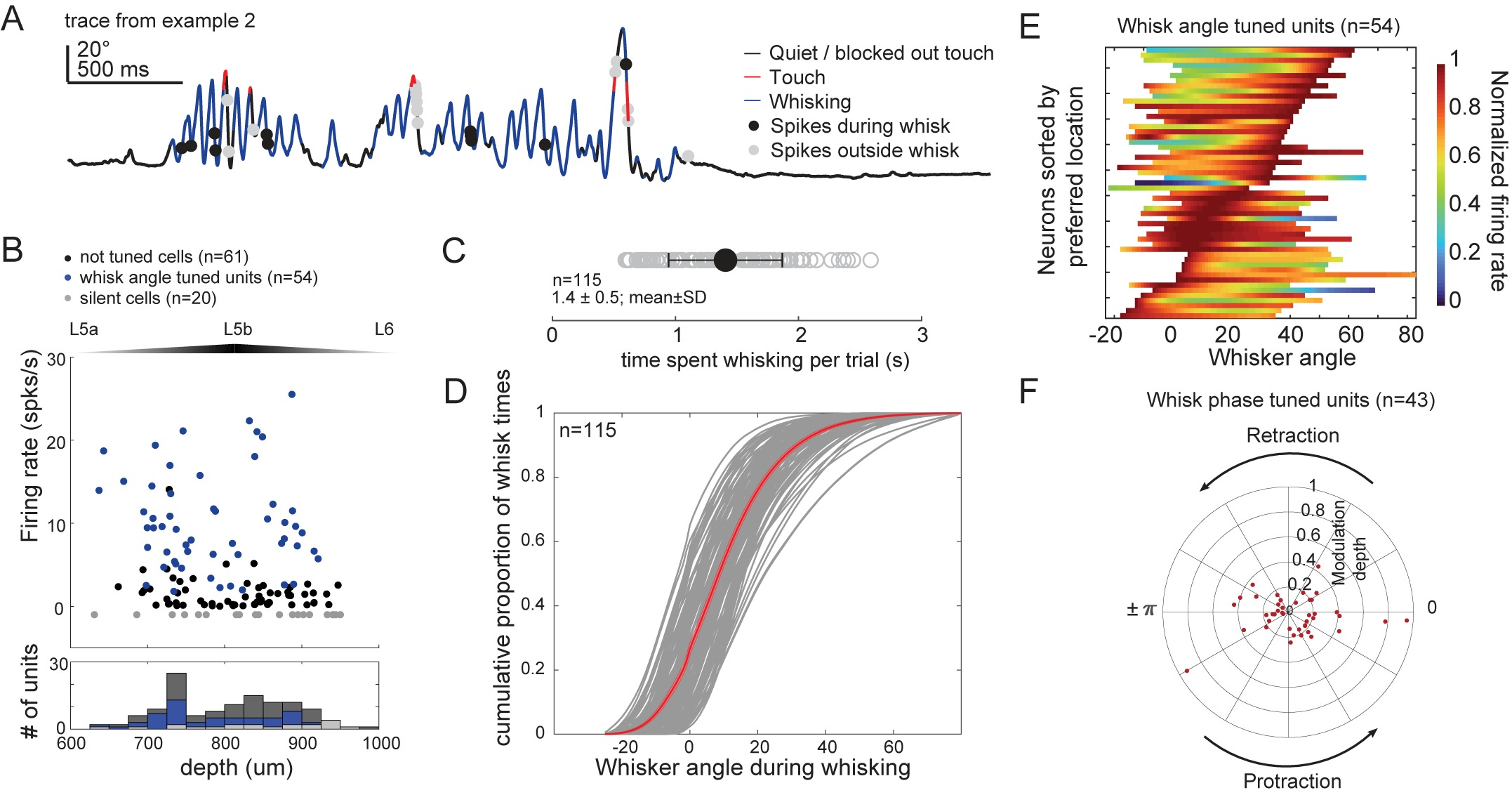

Supplement: S2 Fig — (A) Example whisker trace with spikes overlaid for one example cell tuned to whisker angle during free-whisking. (B) Average firing rate and depth from pia for active non-location (black), whisking angle (blue), and silent (gray) units. (C) Scatter of mean ± SD (1.4 ± 0.5) for time (seconds) spent whisking for each recorded neuron. (D) Cumulative distribution function of whisker-angle sampling during free-whisking for all recorded units (gray) and population average ± SEM (red). (E) Free-whisking angle tuning across the population of significantly tuned units (n = 54). (F) Phase preference with modulation depth (Materials and methods) across the population of phase-tuned units (n = 43). (TIF) [file pbio.3000882.s002.tif]

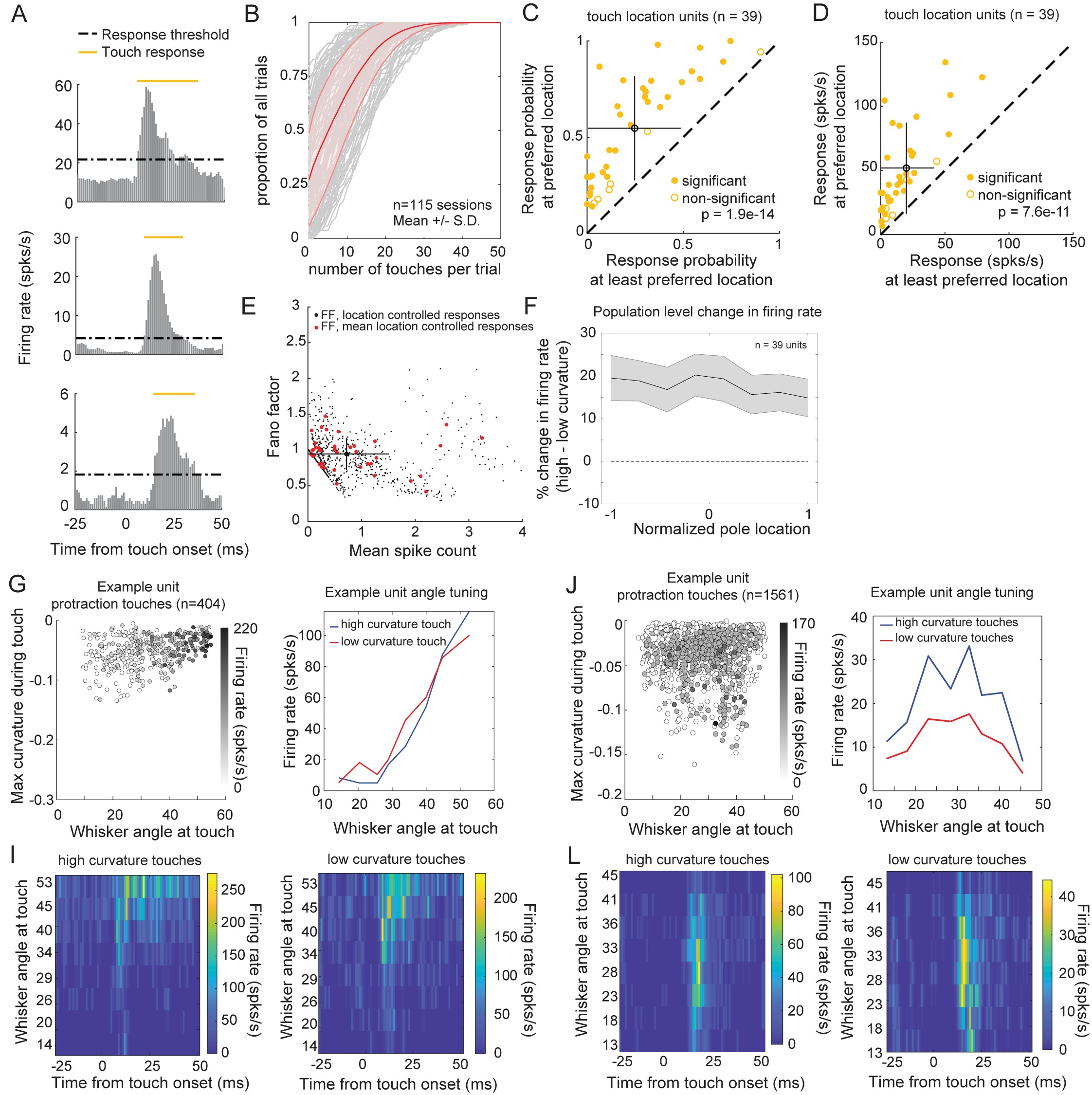

Supplement: S3 Fig — (A) Three example touch units and their responses from touch onset. (B) Cumulative distribution function showing the number of touches made for each recording session (gray, n = 115) and the population average ± SD (red). (C) Response probability of generating a response above baseline ± 95% CI in the most preferred location versus the least preferred location (p = 1.9e-14, t-stat = 11.9, df = 38, paired t test). (D) Same as (C) but for the firing rate of responses (p = 7.6e-11, t-stat = 8.9, df = 38, paired t test). (E) Justification for modeling spikes using a Poisson process. Black dots denote scatter of spike count (0.73 ± 0.81, mean ± SEM) against Fano factor (0.94 ± 0.24, mean ± SEM) for each point along the angle at touch-tuning curve (n = 784 points). Red dots denote average for each individual location at touch-tuned neuron. (F) Population (n = 39 units) difference between high and low curvature touches controlling for position (p = 0.98, one-way ANOVA). (G) Example unit protraction touches (n = 404) highlighting relationship between whisker angle at touch and max curvature of the touch. (H) Single example unit comparing angle-tuning curves for the top 50% of curvature changes (i.e., high) versus the bottom 50% of curvature changes (i.e., low). (I) Heat maps of positional tuning for the same example neuron in (F) and (G) during high (left) and low (right) curvature changes. (J-L) (G), (H), and (I) for another example unit. ANOVA, analysis of variance; max, maximum. (TIF) [file pbio.3000882.s003.tif]

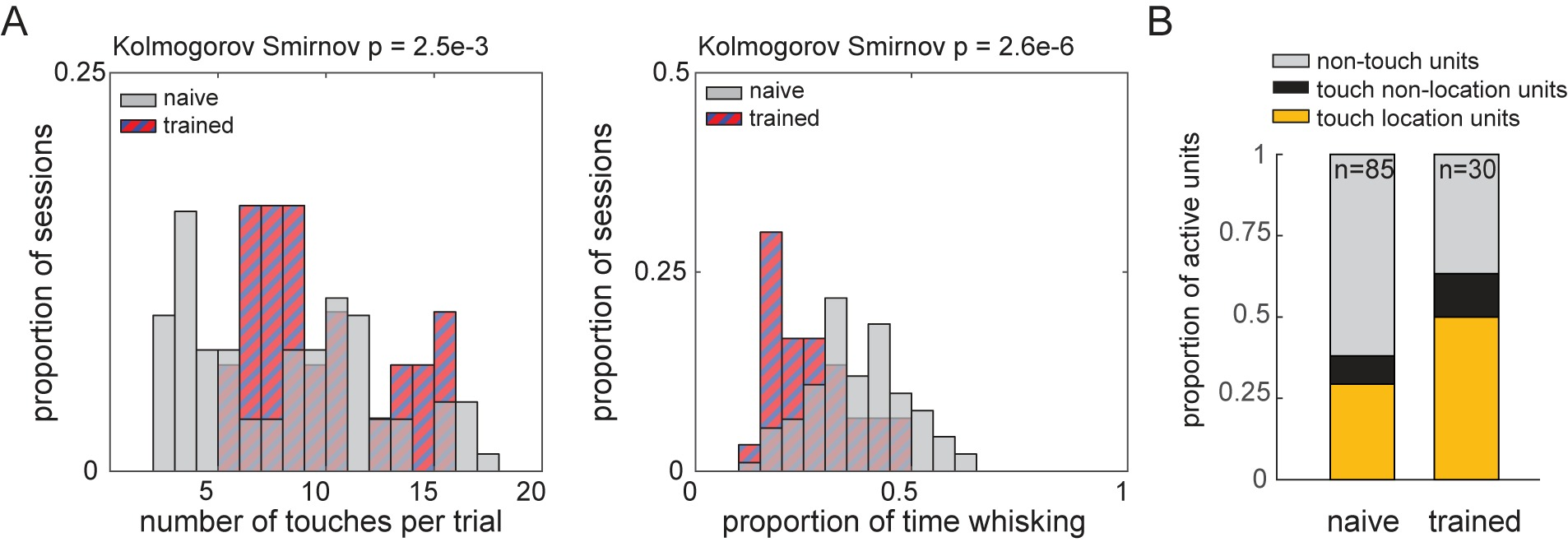

Supplement: S4 Fig — (A) Comparison of number of touches made per trial (left, p = 2.5e-3) and proportion of time whisking (right, p = 2.6e-6) between naïve (gray) and trained animals (red/blue hash). Both compared using two-sample Kolmogorov-Smirnov test. (B) The distribution of non-touch units, touch-location units, and touch non-location units compared between recordings from naïve (n = 85) and trained (n = 30) animals (TIF) [file pbio.3000882.s004.tif]

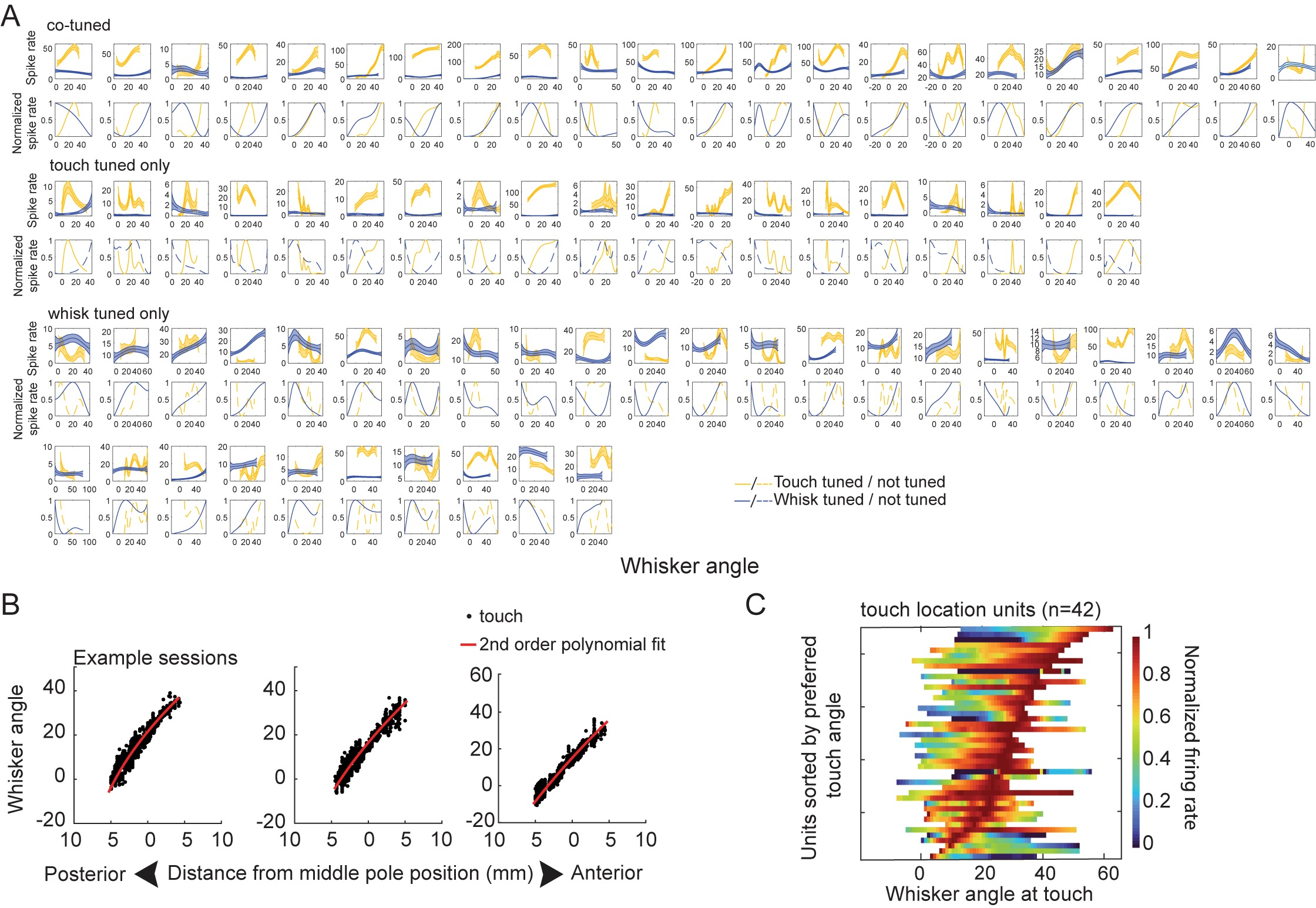

Supplement: S5 Fig — (A) Tuning curves with observed firing rates (top) and normalized firing rates (bottom) for co-tuned (n = 22), touch-tuned only (n = 20), and whisking-tuned only units (n = 32). Solid lines and dashed lines denote tuning and not tuning, respectively. (B) Whisker angle at touch is tightly correlated with anteroposterior object location. Three example sessions are shown. (C) Population heat map of angle-tuned units, sorted by preferred angle at touch. White spaces are insufficiently sampled pole locations. (TIF) [file pbio.3000882.s005.tif]

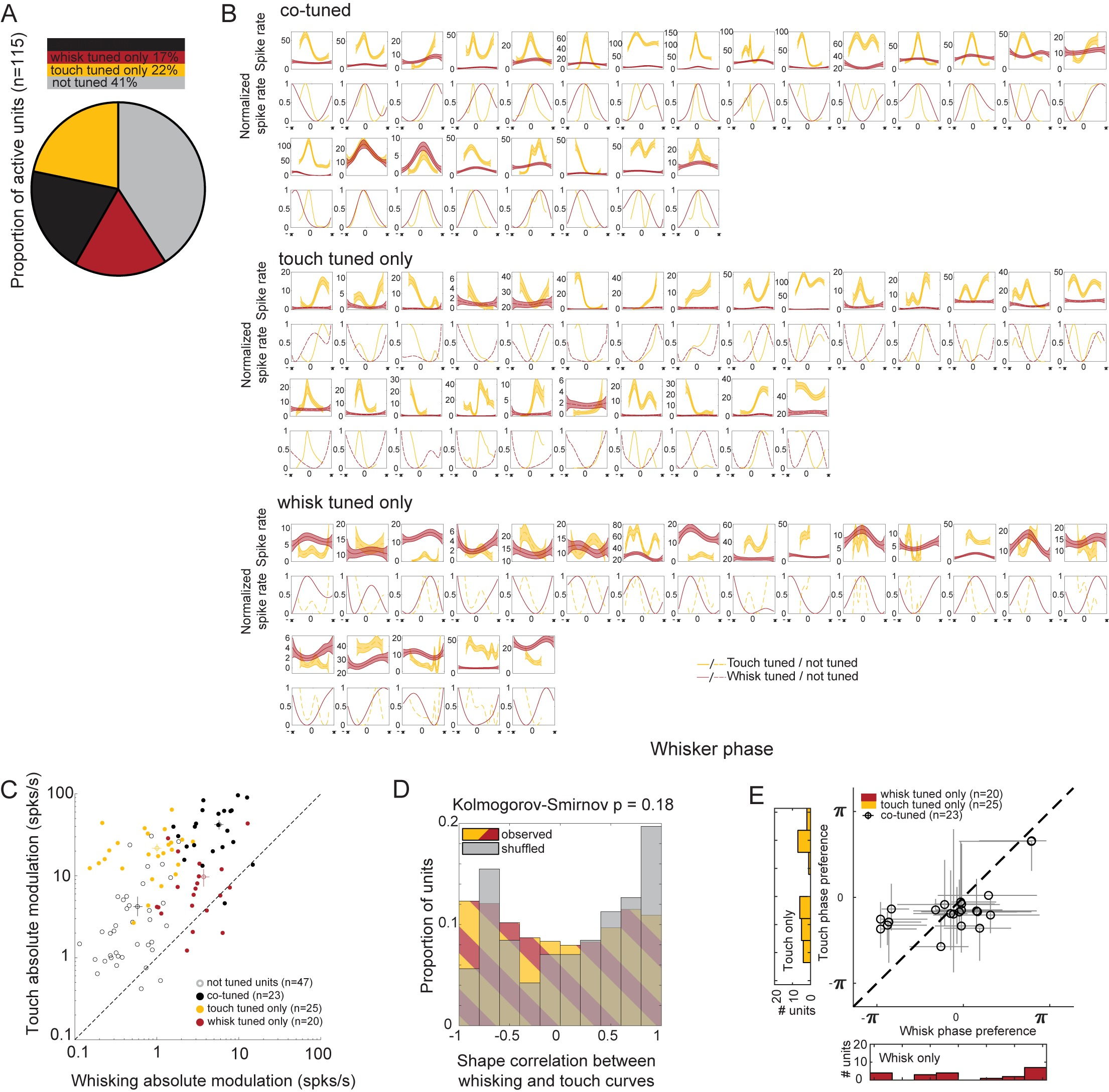

Supplement: S6 Fig — (A) Pie chart highlighting proportion of units phase tuned at whisking (maroon, 20/115), at touch (gold, 25/115), co-tuned (black, 23/115), or not tuned (gray, 47/115). (B) Tuning curves with observed firing rates (top) and normalized firing rates (bottom) for co-tuned (n = 23), touch-tuned only (n = 25), and whisking-tuned only units (n = 20). Solid lines and dashed lines denote tuning and not tuning, respectively. (C) Absolute modulation depth for angle tuning during free-whisking and touch for each class in (A). Average absolute modulation depth was 7× greater for touch (16.5 ± 1.9 Hz; mean ± SEM) than for whisking (2.3 ± 0.3 Hz; mean ± SEM). (D) Shape correlation between whisking- and touch-tuning curves for all units tuned to whisking and/or touch (maroon and gold hash) compared to shuffled responses (gray). Kolmogorov-Smirnov p = 0.11. (E) Scatter of preference during free-whisking and touch for co-tuned units (mean ± SD; 0.7 ± 0.5 radians, p = 1.7e-6, t-stat = 6.4, df = 22; one-sample t test). Histograms denote phase preference for units tuned to either touch or free-whisking phase. (TIF) [file pbio.3000882.s006.tif]
